# Methylated PP2A stabilizes Gcn4 to enable a methionine-induced anabolic program

**DOI:** 10.1074/jbc.RA120.014248

**Published:** 2021-01-13

**Authors:** Adhish S. Walvekar, Ganesh Kadamur, Sreesa Sreedharan, Ritu Gupta, Rajalakshmi Srinivasan, Sunil Laxman

**Affiliations:** 1 Institute for Stem Cell Science and Regenerative Medicine (inStem), Bangalore, India; 2 School of Chemical and Biotechnology, SASTRA University, Tanjavur, India

**Keywords:** methionine, Gcn4, S-adenosylmethionine (SAM), protein phosphatase 2 (PP2A), ATF4, amino acid, nucleotide metabolism, anabolism, cell growth, Saccharomyces cerevisiae, protein methylation, methyltransferase, nucleotide

## Abstract

Methionine, through *S*-adenosylmethionine, activates a multifaceted growth program in which ribosome biogenesis, carbon metabolism, and amino acid and nucleotide biosynthesis are induced. This growth program requires the activity of the Gcn4 transcription factor (called ATF4 in mammals), which facilitates the supply of metabolic precursors that are essential for anabolism. However, how Gcn4 itself is regulated in the presence of methionine is unknown. Here, we discover that Gcn4 protein levels are increased by methionine, despite conditions of high cell growth and translation (in which the roles of Gcn4 are not well-studied). We demonstrate that this mechanism of Gcn4 induction is independent of transcription, as well as the conventional Gcn2/eIF2α-mediated increased translation of Gcn4. Instead, when methionine is abundant, Gcn4 phosphorylation is decreased, which reduces its ubiquitination and therefore degradation. Gcn4 is dephosphorylated by the protein phosphatase 2A (PP2A); our data show that when methionine is abundant, the conserved methyltransferase Ppm1 methylates and alters the activity of the catalytic subunit of PP2A, shifting the balance of Gcn4 toward a dephosphorylated, stable state. The absence of Ppm1 or the loss of the PP2A methylation destabilizes Gcn4 even when methionine is abundant, leading to collapse of the Gcn4-dependent anabolic program. These findings reveal a novel, methionine-dependent signaling and regulatory axis. Here methionine directs the conserved methyltransferase Ppm1 via its target phosphatase PP2A to selectively stabilize Gcn4. Through this, cells conditionally modify a major phosphatase to stabilize a metabolic master regulator and drive anabolism.

Cellular commitments to different states, such as growth, survival, or self-destruction, depend on multiple cues. The availability of nutrients is a critical signal for cells to switch to a growth state. When adequate supplies of carbon and nitrogen are available, cells channel them into energy production, biomass generation, and ribosomal biogenesis. These processes collectively lead to cell growth and division. Using simple model organisms like yeast, systems-level studies have investigated cellular responses to distinct nutrient availabilities and defined transcriptional programs that reflect growth or starvation states (1, 2, 3, 4, 5, 6). When cells commit to mitotic division, multiple signaling cascades and transcriptional responses control such “growth programs” (1, 3, 6, 7, 8, 9, 10). Although global responses to nutrient changes are well-documented, specific signaling and regulatory mechanisms that directly couple the sensing of specific metabolites to metabolic programs remain poorly studied. Thus, there is a need to identify these regulatory mechanisms, to mechanistically understand growth programs.

In this regard, some metabolites directly act as “sentinel” molecules that trigger growth programs. One such metabolite that signals a multifaceted growth program is methionine (11). Studies using yeast cells revealed that methionine inhibits autophagy and concomitantly also increases cell proliferation (12, 13, 14). Methionine (via its primary metabolite, SAM) directly activates the target of rapamycin (TOR) complex I in yeast and mammalian cells (13, 15, 16), regulates lipid balance (17), increases translational capacity, and maintains metabolic homeostasis during growth (14). Consistent with these roles, studies from a variety of cancers suggest that methionine is a key determinant of tumor cell proliferation (18, 19, 20, 21). Indeed, a fundamental determinant of cell growth is the steady supply of anabolic precursor molecules, which fuel sufficient translation and RNA/DNA synthesis. Methionine is itself incapable of being a metabolic precursor to these building blocks and instead appears to function as a signaling molecule, activating these processes, likely via SAM. However, the specific signaling events that directly control this metabolic transformation and thereby lead to the continuous supply of anabolic molecules remain unclear.

In a recent study, we discovered that methionine controls a hierarchically organized, well-defined transcriptional program (14). In this program, the addition of methionine induces the expression of genes involved in translation and ribosomal biogenesis, as well as key metabolic reactions leading to anabolism. This primarily comprises the pentose-phosphate pathway, glutamine synthesis, and amino acid and nucleotide biosynthesis. In short, the addition of methionine switched cells to a signature growth state, in which the assimilation and utilization of available carbon and nitrogen sources resulted in increased amino acid and nucleotide biosynthesis required for growth (14, 22). Surprisingly, to sustain this growth program and maintain the supply of anabolic precursors, cells required the activity of what is primarily considered a starvation/survival response transcription factor: Gcn4 (22). How Gcn4 can be induced by methionine remains a mystery.

Gcn4 (called ATF4 in mammals), is a well-studied transcriptional activator of genes in amino acid biosynthetic pathways (23, 24). Under amino acid starvation conditions, Gcn4 has a key role in restoring amino acid homeostasis (24, 25, 26, 27). When cells are starved of even a single amino acid, the translation of Gcn4/ATF4 transcripts increases in a Gcn2/phospho-eIF2α–dependent manner. During general amino acid starvation, the Gcn2 protein kinase is activated, down-regulating global translation with a concomitant enhancement of Gcn4 translation. This promotes the expression amino acid biosynthetic gene transcripts, enabling cells to restore amino acid pools (24). Paradoxically, our findings (14, 22) are noteworthy because Gcn4 was critical in a context of high growth and proliferation, rather than survival, when methionine alone was supplemented. Therefore, in such a context of *increased* proliferation, we asked what signaling and regulatory processes control Gcn4 induction.

In this study, we elucidate a novel mechanism through which Gcn4 protein is induced during this methionine-dependent growth program. Specifically, abundant methionine does not additionally enhance Gcn4 transcription or translation. Instead, methionine post-translationally increases Gcn4 protein via a SAM-dependent methylation of the protein phosphatase PP2A by the methyl transferase Ppm1 (LCMT1 in mammals). Methylated PP2A maintains Gcn4 in a dephosphorylated state. This prevents the phosphorylation-dependent ubiquitination of Gcn4 that typically leads to subsequent proteasomal degradation. The stabilization of Gcn4 is not observed in the absence of Ppm1 or upon the loss of PP2A methylation. Further, this PP2A activity is required for Gcn4-mediated high *de novo* amino acid and nucleotide biosynthesis. Our findings illustrate how a methionine/SAM-responsive protein phosphatase, PP2A, directly controls anabolism by increasing amounts of a metabolic master regulator, Gcn4. This reveals how a methionine-responsive signaling response can coordinately control the metabolic state of the cell.

## Results

### Gcn4 is induced by methionine while cells retain a high translation state

In yeast cells growing in overall amino acid limited conditions with lactate as a carbon source, supplementing methionine drives a transcriptional and metabolic growth program (14, 22). Methionine transcriptionally induces rate-limiting reactions in the pentose phosphate pathway and amino acid biosynthesis, which functionally increases amino acid and nucleotide synthesis (14). Methionine also transcriptionally induces ribosomal genes and ribosome biogenesis, suggesting that it up-regulates overall protein synthesis (14). In these conditions, methionine (via SAM) also activates the primary eukaryotic growth pathway, the TORC1 pathway, while inhibiting autophagy (13). Collectively, these studies suggest that methionine functions as a growth signal.

**Figure 1:**
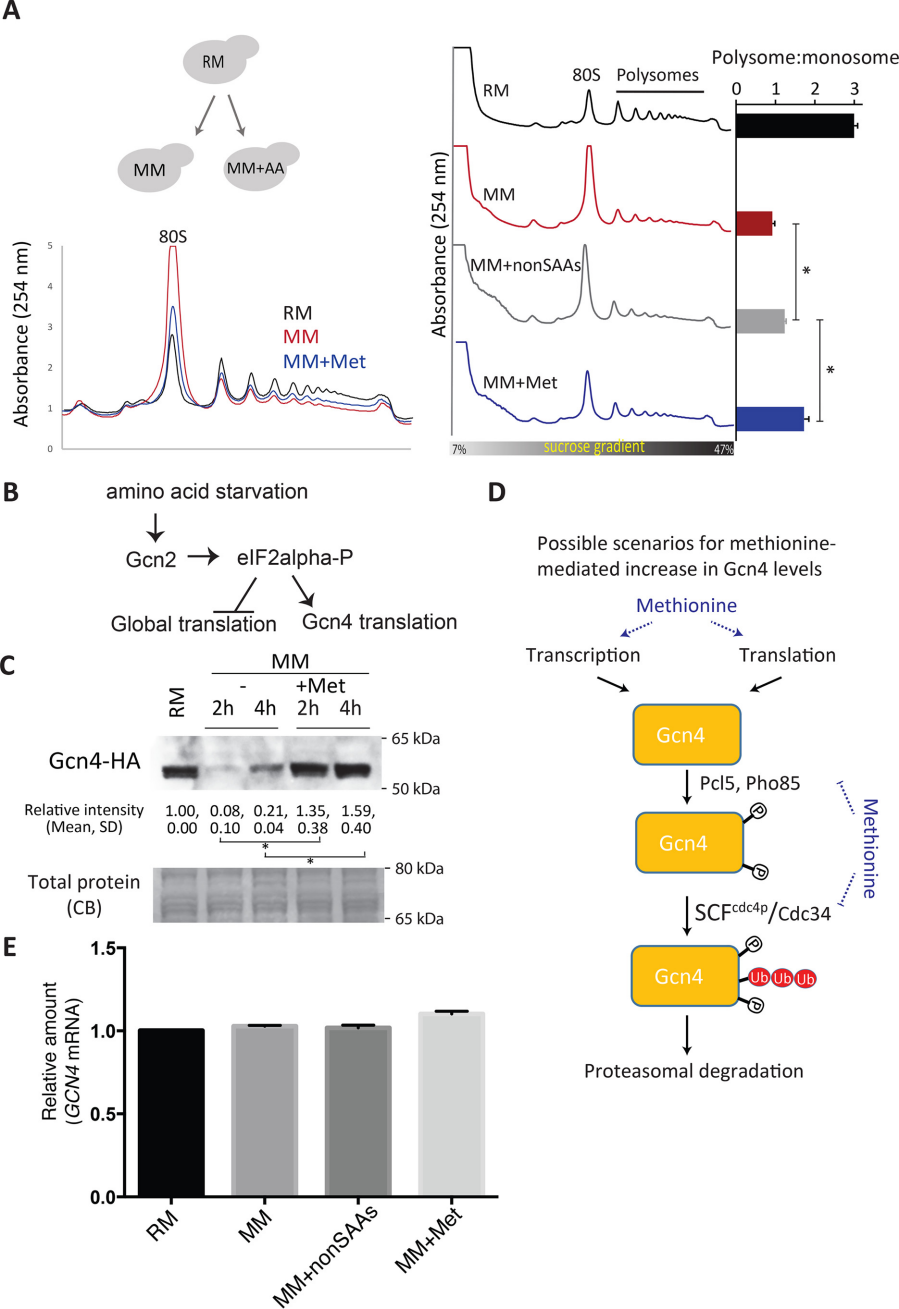
**Gcn4 is induced by methionine while cells are in a high translation state.***A*, polysome profiles and polysome:monosome ratios of cells growing in RM, MM, or MM + Met. The plots shown are from three biological replicates (means ± S.D). *, *p* < 0.05 (Student's *t* test). Also see Fig. S1. *B*, a canonical model of Gcn4 induction mediated via the increased translation of Gcn4 transcripts and controlled by the activation of the Gcn2 kinase and the phosphorylation of eIF2α. *C*, methionine-dependent Gcn4 induction. Gcn4 protein amounts were estimated in cells growing in RM, MM, or MM + Met, as measured by Western blotting. Relative Gcn4 amounts in each indicated condition were quantified based on band intensity, and quantifications are indicated *below* the blot (means ± S.D.). *, *p* < 0.05 (Student's *t* test). Each experiment was performed in biological replicates. *D*, plausible scenarios for how methionine might increase Gcn4 levels. We consider possible increases in Gcn4 caused by increased transcription and translation, translation alone, or reduced degradation (including the known mechanisms leading to Gcn4 degradation). *E*, relative amounts of Gcn4 transcripts in RM, MM, and MM + Met. Data shown are from whole-transcriptome (RNA sequencing) data from biological replicates, obtained from Ref. 14. Differences in Gcn4 transcript amounts between any two of the different conditions are statistically not significant (Student's *t* test).

To confirm that this transcriptional induction of ribosomal genes was functionally observed as increased translation, we examined the impact of methionine addition on global translation using polysome profiling. Expectedly, yeast cells grown in a complex, rich medium (RM) showed a 3:1 polysome:monosome ratio indicating robust translation in the system (Fig. 1*A*). In contrast, cells shifted to a defined minimal medium with the same carbon source (MM) showed a drastic reduction in the polysome pool and an increased monosome peak (polysome:monosome ratio of ∼1:1), reflective of low translation (Fig. 1*A*). Methionine supplementation during a shift to MM medium (MM + Met) significantly restored polysome levels, resulting in a polysome:monosome ratio of ∼1.7:1 (Fig. 1*A*). This increase in polysome levels is greater than that observed with supplementing all nonsulfur amino acids combined except tyrosine (pool of 17 amino acids, excluding methionine and cysteine). These data therefore confirm that methionine supplementation increases global translation in otherwise amino acid limited conditions. These data are entirely consistent with the predictions from the observed transcriptional signatures of a growth program induced by methionine (14), as well as increased growth when methionine is added (13).

While dissecting the transcriptional program that methionine controls, we had unexpectedly uncovered a critical role for Gcn4 (14, 22). Methionine strongly increases growth and induces amino acid and nucleotide biosynthesis (14). Both of these processes were entirely Gcn4-dependent. Cells lacking Gcn4 did not transcriptionally or functionally induce these metabolic pathways, which are critical to fuel the growth in these still limiting conditions (14, 22). Further, the ability of cells to maintain high translation in these conditions was Gcn4-dependent. The loss of Gcn4 resulted in a collapse of the high translation in these cells, because of a limiting supply of critical amino acids (22).

This was an unexpected observation, for the following reasons. Gcn4 is a transcription factor that has been best studied as an activator of amino acid biosynthetic genes during severe amino acid starvation (24, 25, 26, 28, 29, 30). During starvation conditions (including methionine starvation), the Gcn2 kinase phosphorylates eIF2α, resulting in a damping of global translation with simultaneous increase in Gcn4 levels (Fig. 1*B*) (24, 31). This induction of Gcn4 enables cells to restore amino acid amounts. The observations made in methionine-replete conditions therefore posted a paradox. Here, global translation is clearly increased (Fig. 1*A*), yet Gcn4 function is critical under these conditions. Hence, our primary objective was to address how abundant methionine might regulate Gcn4.

Using a strain in which a C-terminal hemagglutinin (HA) tag was inserted at the chromosomal *GCN4* locus, we first measured steady state levels of Gcn4 protein after a shift from RM to MM. As observed earlier (14, 22), Gcn4 levels were high only in cells shifted from robust growth conditions (RM) to MM + Met (Fig. 1*C*). In contrast, cells shifted to medium with no amino acids (MM) had comparatively low Gcn4 amounts (Fig. 1*C*), as did cells shifted to medium where all nonsulfur amino acids were supplemented except tyrosine (MM + non-SAA), as also shown earlier (14). This reiterated the above-stated paradox: when methionine is abundant and the cells are in a state of high growth and translation, Gcn4 amounts are also strongly induced. Therefore, how might abundant methionine increase Gcn4 levels?

### Gcn4 accumulation caused by methionine does not depend on the GCN2-translation axis

Gcn4 protein levels can be fine-tuned through possible transcriptional, translational, or post-translational mechanisms (Fig. 1*D*). We investigated which of these steps is under methionine regulation. For this, we first compared mRNA levels of *GCN4* in medium with and without methionine supplementation, using RNA-sequencing data sets from our previous study (14). *GCN4* transcript levels were not significantly higher in MM + Met compared with that in minimal medium lacking all amino acids (Fig. 1*E*). Transcript levels of *GCN4* in medium supplemented with all amino acids except methionine/cysteine was also similar in this data set (14). Thus, *GCN4* expression is not transcriptionally induced by methionine.

**Figure 2:**
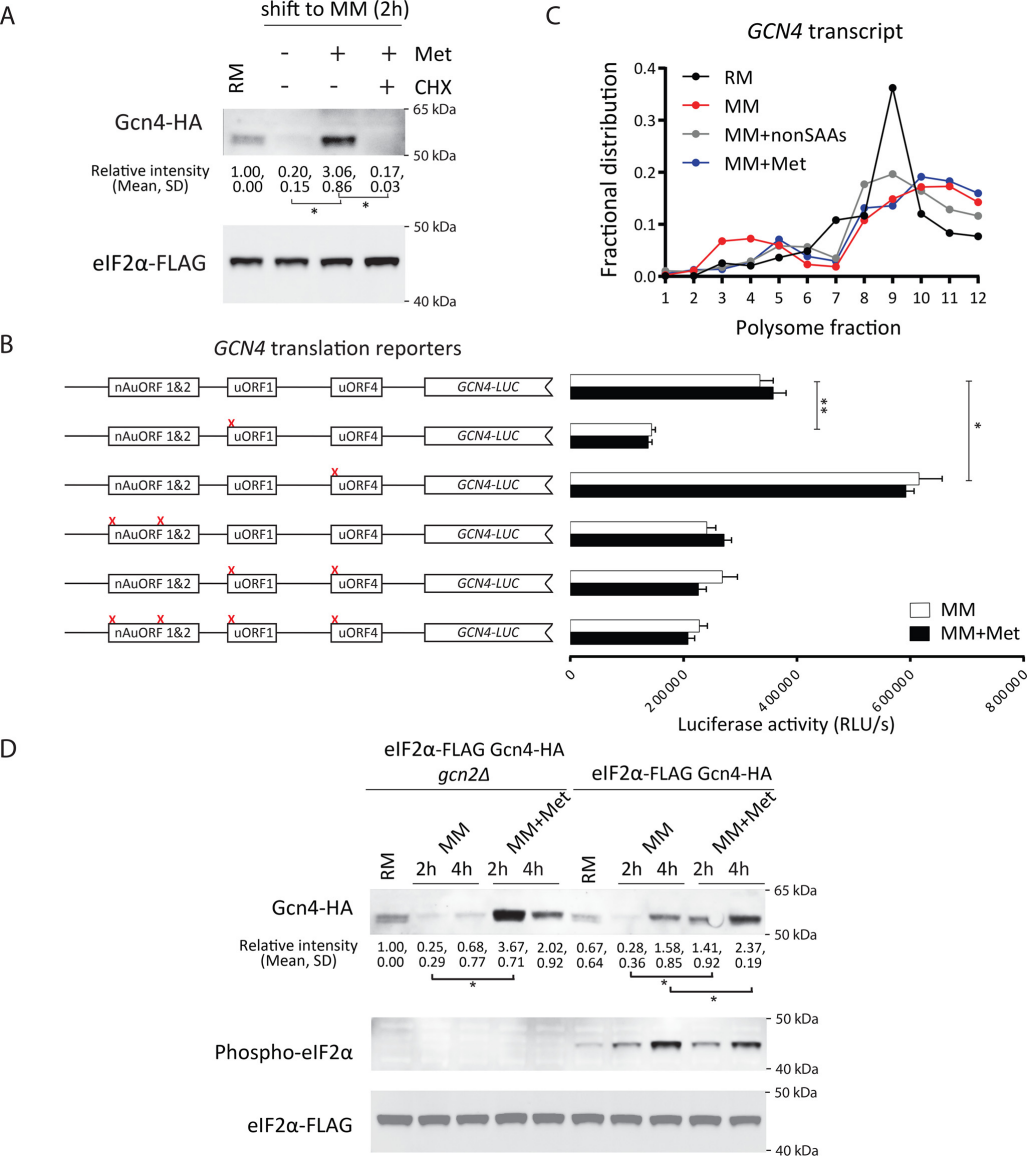
**Methionine-induced Gcn4 accumulation does not depend on the GCN2-translation axis.***A*, effect of cycloheximide treatment on Gcn4 protein amounts. Cells growing in MM or MM + Met were treated with cycloheximide (*CHX*), and Gcn4 protein amounts were estimated by Western blotting. Relative Gcn4 amounts in the indicated conditions were quantified based on band intensity, and quantifications are indicated *below* the blot (means ± S.D.). *, *p* < 0.05 (Student's *t* test). Each experiment was performed in biological replicates. *B*, relative *GCN4* translation in MM or MM + Met, as measured using a series of luciferase-based *GCN4* translation reporters. The relative luciferase activity is shown on the *y* axis of the plots, whereas the different reporters used are illustrated on the *left*. The data shown are from three biological replicates (means ± S.D.). *, *p* < 0.05; **, *p* < 0.01 (Student's *t* test). Also see Fig. S1 (A–C). *C*, *GCN4* transcript amounts in different polysome fractions, obtained from cells grown in RM, MM, or MM + Met. Transcripts were measured using standard, quantitative RT-qPCR approaches. Also see Fig. S1D. *D*, Gcn4 protein amounts estimated in WT or *gcn2*Δ cells, grown in MM or MM + Met. The yeast ortholog of eIF2α (Sui2) was also tagged with a FLAG epitope (in the native chromosomal locus). As controls, the amounts of phosphorylated eIF2α (as detected using a phospho-specific eIF2α antibody) and total eIF2α protein are shown. Total or phosphorylated forms of eIF2α amounts did not change across MM and MM + Met in WT cells, and phosphorylated forms of eIF2α were not detectable in *gcn2*Δ cells. Relative Gcn4 amounts in the indicated conditions were quantified based on band intensity, and quantifications are indicated *below* the blot (means ± S.D.). *, *p* < 0.05 (Student's *t* test). Each experiment was performed in biological replicates. Also see Fig. S2.

We therefore compared the relative rates of synthesis of Gcn4. To test whether Gcn4 is actively synthesized after methionine addition, the cells were treated with cycloheximide, a commonly used ribosome inhibitor. Gcn4 amounts were reduced when cells were shifted to methionine-replete medium supplemented with cycloheximide (Fig. 2*A*). This indicates that Gcn4 is continuously, actively translated under these conditions.

The post-transcriptional regulation of Gcn4 is complex and achieved by multiple modes (24). The most extensively studied regulation is through the control of *GCN4* translation during amino acid starvation. During starvation the translation of *GCN4* transcripts increases. This is mediated by the activated Gcn2 kinase, which phosphorylates eIF2α (24, 32, 33). This *GCN4* translation is dictated by its 5′ leader sequence (Fig. 2*B*), primarily by the upstream ORF 1 (uORF1) and uORF4, as well as through two non-AUG uORFs that are present upstream of the uORFs (24, 34, 35, 36, 37, 38, 39, 40). We therefore investigated whether these mechanisms that increase the translation of *GCN4* might be relevant in the presence of methionine. For this we utilized a series of standard *GCN4*-luciferase fusion reporters. These reporters report on the extent of *GCN4* translation mediated by these various regulatory elements, as established earlier (40, 41). We specifically asked whether these regulatory features might contribute to the methionine-specific enhancement of *GCN4* translation. A series of *GCN4*-luciferase translation reporters with point mutations in each of these known regulatory elements were therefore included. All constructs were first thoroughly validated using a conventional method of *GCN4* translation activation upon rapamycin addition, and the reporters all functioned as would be expected (Fig. S1, A and B). Using these reporters, we compared the translation of *GCN4* in minimal medium with or without methionine supplementation (Fig. 2*B*) or in medium with all amino acids except methionine and cysteine (Fig. S1C). Notably, the activity of these reporters was comparable and high in both MM and MM + Met (Fig. 2*B* and Fig. S1C). This indicates that the translation of the *GCN4* transcript in both these conditions (MM and MM + Met) remains continuously high. Importantly, the luciferase reporter activity was not significantly further enhanced in the presence of methionine, indicating that the translation rate of the *GCN4* ORF is persistently high in minimal medium regardless of methionine addition. To independently validate this, the distribution of native *GCN4* transcript was measured in polysome fractions of cells shifted to MM medium with or without amino acid supplementations. Consistent with the reporter data, we found no notable change in the *GCN4* transcript distribution in any of the combinations tested, particularly MM and MM + Met (Fig. 1*C* and Fig. S1D). Collectively, these data reveal that GCN4 is actively translated at comparable rates in MM medium regardless of methionine supplementation.

These data therefore also suggested that the resulting Gcn4 accumulation in methionine might be independently regulated beyond the Gcn2-eIF2α regulatory axis. To test this, we asked whether the increased Gcn4 amounts seen in the presence of methionine depended on Gcn2 activity. Even in a *gcn2*Δ strain, Gcn4 accumulated upon methionine supplementation (Fig. 2*D*), suggesting that other mechanisms contribute to the methionine-mediated accumulation of Gcn4. Finally, the proliferation of WT, *gcn2*Δ, and *gcn4*Δ cells were compared in MM + Met. Notably, in this condition, *gcn2*Δ grew significantly better (partial growth rescue) than *gcn4*Δ cells in MM + Met (Fig. S2). *gcn4*Δ cannot grow in MM + Met (Fig. S2), as also observed earlier (14). As controls, the growth of cells from these genetic backgrounds were compared in complete RM, where no significant growth defect could be observed (Fig. S2). This decrease in growth, as well as the decrease of Gcn4 in the *gcn2*Δ strain, is consistent with the role of Gcn2 in maintaining synthesis but not Gcn4 stability or turnover. Therefore, the canonical mechanism of *GCN4* control at the translational level by the GCN2–eIF2α axis cannot fully explain the increased Gcn4 levels specifically in the presence of methionine. Taken together, the above results suggest the presence of alternative post-translational mechanisms that may regulate Gcn4 levels.

### Methionine inhibits Gcn4 degradation by the 26S proteasome

**Figure 3:**
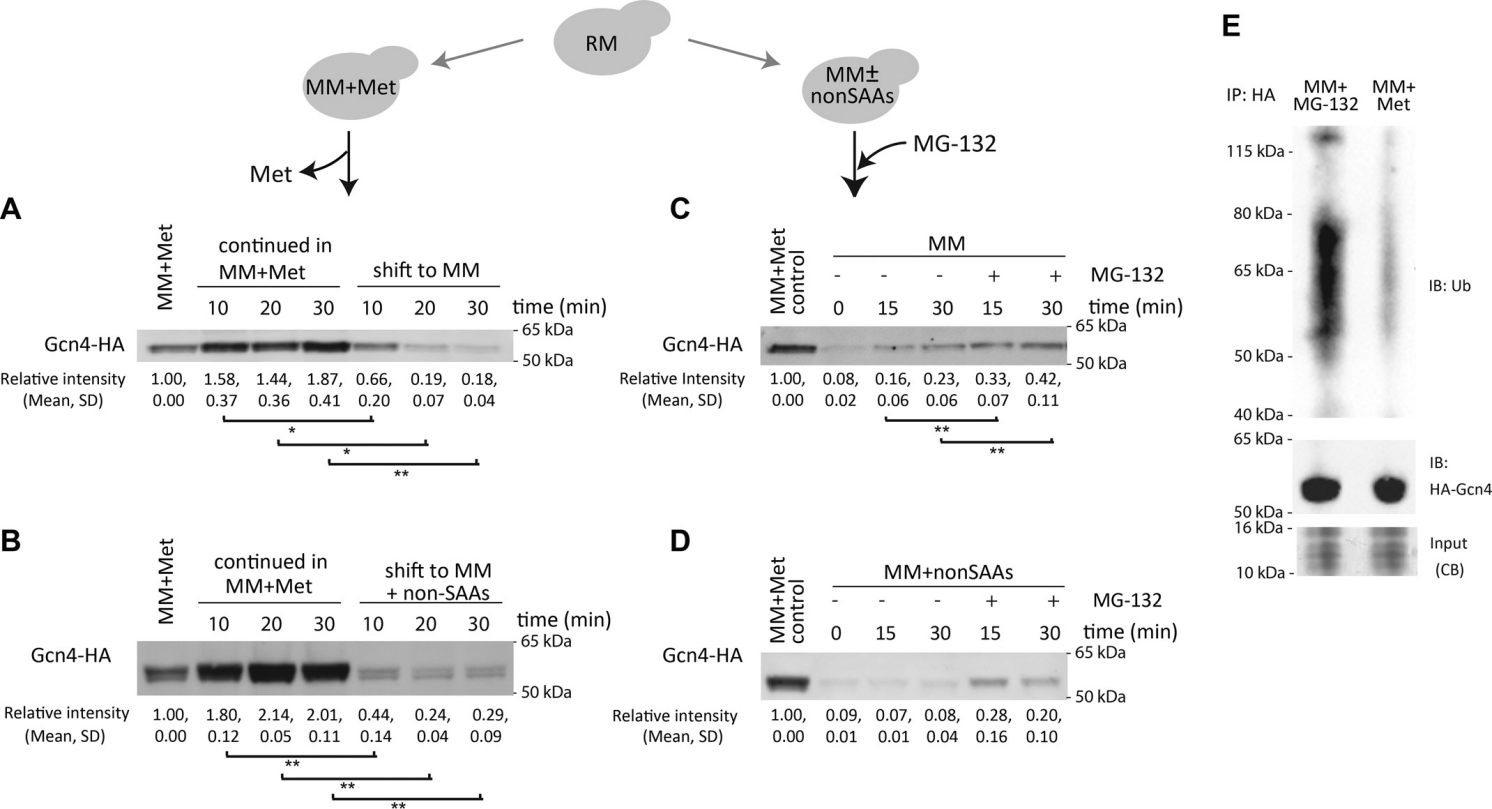
**Methionine inhibits Gcn4 degradation.***Top schematic*, the experimental design used to address Gcn4 stability and kinetics of degradation in the absence of methionine. *A*, cells growing in MM + Met were shifted to MM, and Gcn4 protein amounts were estimated from cells collected from across a short time course. Notably, Gcn4 amounts decrease substantially within 10 min of shift to MM. *B*, cells growing in MM + Met were shifted to MM + all non-SAAs, and Gcn4 protein amounts were estimated across a short time course. Notably, Gcn4 amounts decrease substantially within 10 min of shift to MM. *C*, proteasomal degradation of Gcn4 in the absence of methionine. Cells growing in MM + Met were shifted to MM in the presence or absence of the proteasomal inhibitor MG132, and Gcn4 protein amounts were estimated across a short time course. Also see Fig. S3. *D*, cells growing in MM + Met were shifted to MM + non-SAA in the presence or absence of the proteasomal inhibitor MG132, and Gcn4 protein amounts were estimated across a short time course. Gcn4 amounts accumulate in the presence of MG132. For *A–D*, the relative Gcn4 amounts in the indicated conditions were quantified based on band intensity, and quantifications are indicated *below* the blot (means ± S.D.). *, *p* < 0.05; **, *p* < 0.01 (Student's *t* test). Each experiment was performed in biological replicates. For each lane, the same amount of total protein was loaded. For only Fig. 3*D*, quantifications when compared were not statistically significant (*p* > 0.05). *E*, Gcn4 ubiquitination decreases in the presence of methionine. Cells growing in MM (with MG132 added) or MM + Met were collected, and Gcn4 was immunopurified. The immunopurified Gcn4 was resolved on SDS-PAGE gels, and polyubiquitin chains were detected by Western blotting using an ubiquitin-specific antibody. Cells in MM + Met show substantially decreased polyubiquitin bands. *IB*, immunoblot; *IP*, immunopurification; *CB*, Coomassie Blue stain. Gel representative of two biological replicates (also see Fig. S4).

Although the regulation of Gcn4 has been more extensively investigated at the level of its synthesis, this can also occur at the level of degradation (42), as illustrated in Fig. 1*D*. The targeted degradation of Gcn4 involves its polyubiquitination and subsequent degradation by the 26S proteasome. We therefore asked whether methionine might reduce the proteasomal degradation of Gcn4. For this, we devised a modified experimental setup in which the role of methionine in Gcn4 degradation could be specifically examined (Fig. 3). We first asked how rapidly Gcn4 amounts decreased in cells, when shifted from methionine-replete medium (MM + Met) to methionine-limited medium (MM). For this, we allowed Gcn4 to accumulate in MM + Met and then measured Gcn4 protein levels after shifting the cells to medium lacking methionine. Indeed, Gcn4 protein levels rapidly decreased within 20 min of shift of cells to MM (Fig. 3*A*) or a shift to MM + non-SAAs (where free methionine is not present) (Fig. 3*B*). Given this rapid decrease in Gcn4 amounts, along with the earlier data showing that Gcn4 translation remains high in MM (Fig. 2), these observations are consistent with a possible role for methionine in stabilizing Gcn4 by modulating its degradation.

We next tested whether the decreased Gcn4 amounts observed in MM (*i.e.* after methionine removal) could be reversed by inhibiting proteasomal activity. Here, the rapid degradation of Gcn4 was inhibited upon the addition of the proteasome inhibitor MG-132 to cells in MM or MM + non-SAAs (Fig. 3, *C* and *D*). These results suggest that in amino acid limited conditions, the availability of methionine inhibits Gcn4 degradation. Note that in yeast it is challenging to use MG-132 to transiently inhibit the proteasome, especially in these specific MM/MM + non-SAAs conditions in which autophagy is induced (13). To facilitate some retention of MG-132, experiments were done in the *pdr5*Δ background (43). We also ensured that changes in Gcn4 were not due to the addition of small amounts of DMSO (in which MG-132 was dissolved) (Fig. S3).

Next, we examined the ubiquitination status of Gcn4 and its modulation by methionine. Because ubiquitinated species are rapidly turned over by the 26S proteasome, MG-132 was added to inhibit degradation and enable some accumulation of polyubiquitin-conjugated proteins. Replicating the earlier setup, the cells were initially adapted to MM + Met (to allow Gcn4 accumulation) and then shifted them to MM in the presence of either methionine or MG-132. Subsequently, polyubiquitin chains were detected on immunopurified Gcn4 (Fig. 3*E* and Fig. S4). Immunoblot analysis showed a smear of high-molecular-weight, ubiquitinated Gcn4 only in cells shifted to medium lacking methionine (Fig. 3*E* and Fig. S4). However, such ubiquitinated species were almost undetectable in cells that had remained in MM + Met (Fig. 3*E*). These data collectively suggest that although Gcn4 is constantly synthesized in MM regardless of methionine availability, abundant methionine specifically inhibits the ubiquitination and proteasomal degradation of Gcn4.

### Methionine does not alter the composition of the Gcn4 targeting ubiquitination machinery

**Figure 4:**
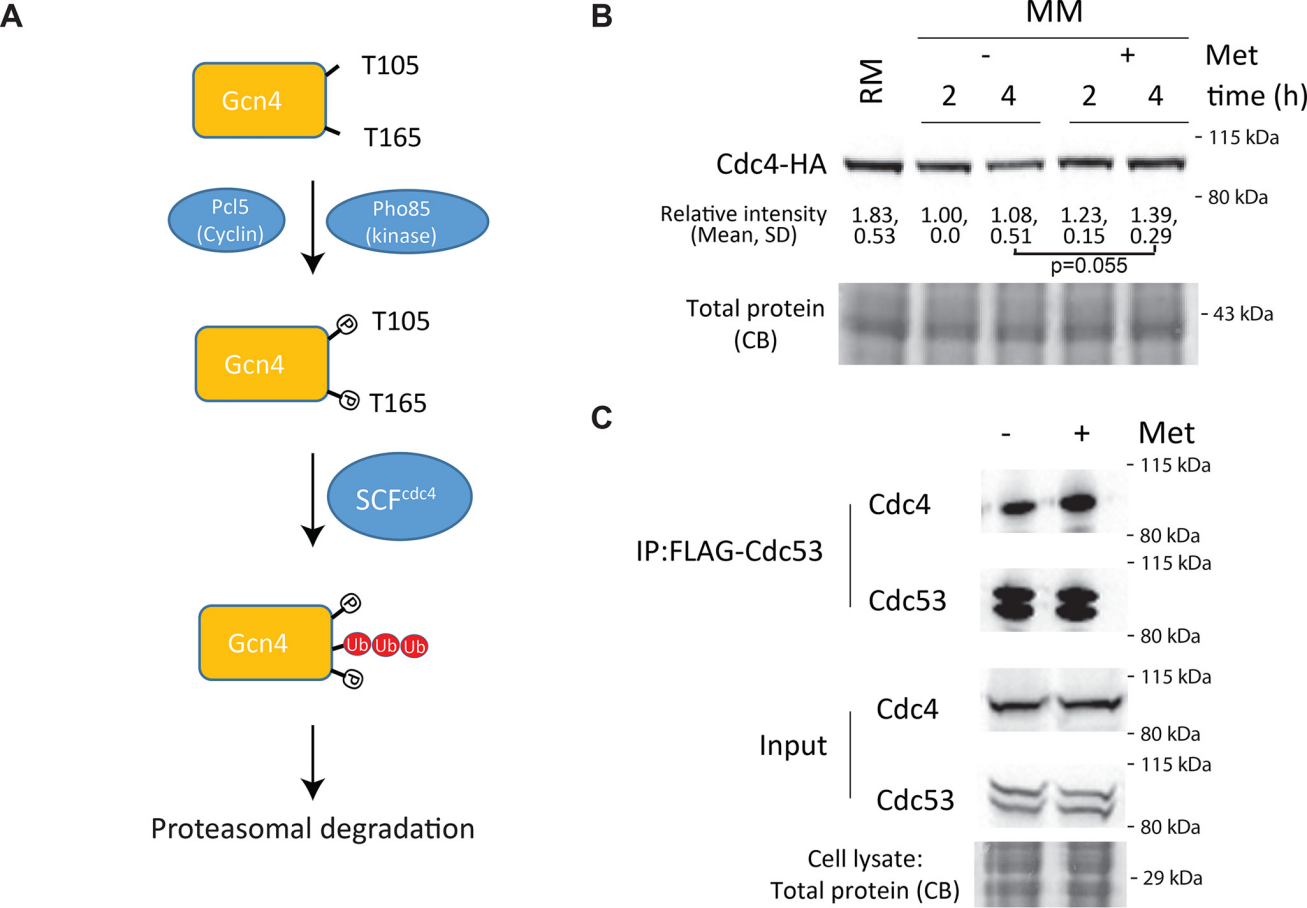
**Methionine does not decrease the amounts of or composition of the Gcn4 targeting ubiquitination machinery.***A*, a schematic illustration indicating known mechanisms of Gcn4 degradation. Gcn4 can be phosphorylated (dependent on the Pho85 kinase), and this phosphorylation of two threonine residues leads to the ubiquitination of Gcn4 by the SCF complex (which includes Skp1, Cdc53, and the specific F-box Cdc4). This leads to Gcn4 degradation. *B*, relative amounts of Cdc4 in MM or MM + Met. Cells in RM were shifted to MM or MM + Met, and Cdc4 amounts were measured by Western blotting. Relative protein amounts in the indicated conditions were quantified, and the quantifications are indicated *below* the blot (means ± S.D.). *, *p* < 0.05 (Student's *t* test). Each experiment was performed in biological replicates. *C*, interaction of Cdc53 and Cdc4, and the formation of the SCF^CDC4^ complex. Cells in MM or MM + Met were collected, Cdc53 (FLAG) was immunoprecipitated (*IP*), and the samples were subjected to HA immunoblots to assess the association between Cdc4 and Skp1–Cdc53 complex. Relative amounts of the respective proteins in the indicated conditions are quantified and indicated *below* the blot (means ± S.D.). *, *p* < 0.05 (Student's *t* test). Each experiment was performed in biological replicates. Also see Fig. S5. *CB*, Coomassie Blue stain.

The ubiquitination of Gcn4 is known to be carried out by the (SCF) family of ubiquitin E3 ligases (42, 44) (Fig. 4*A*). Skp1 and Cullin are evolutionarily conserved proteins that form a stable complex, whereas the F-box is a variable component that defines the target specificity (45). Cdc4 is a F-box protein and part of the SCF^Cdc4^ complex that includes Skp1 and the Cullin protein Cdc53. This complex is required for G_1_/S and G_2_/M cell cycle transitions. In addition to its role in the cell cycle, this protein also ubiquitinates Gcn4 (42, 44). Hence, we first examined whether methionine alters Cdc4 amounts. This in turn could modulate ubiquitination activity toward substrates including Gcn4 in a methionine-specific manner. For this, the relative Cdc4 levels were compared in cells grown in RM, cells shifted to MM, or MM + Met. Cdc4 amounts did not decrease in MM + Met and in fact showed a small increase in amounts compared with MM (Fig. 4*B*). This indicates that Cdc4 is not specifically decreased by methionine availability. We next asked whether methionine instead alters the binding of Cdc4 to Skp1–Cdc53, which could impact the functional SCF^Cdc4^ pool. Using a strain in which both Cdc4 and Cdc53 were chromosomally epitope-tagged, the association of Cdc4 with the Skp1–Cdc53 complex was analyzed in cells that were shifted from RM to either MM or MM + Met. For this, the cells were grown for 2 h post-shift to the respective medium to allow the re-equilibration of cellular SCF complexes. Cdc53 was subsequently immunoprecipitated, and the samples were subjected to immunoblots to assess the association between Cdc4 and Skp1–Cdc53 complex. In both MM and MM + Met, Cdc4 was immunoprecipitated, with a small (but not significant) relative increase in the amount of Cdc4 immunoprecipitated from MM + Met (Fig. 4*C*). Importantly, the addition of methionine did not decrease Cdc4 amounts or decrease the association of Cdc4 with the Skp1–Cdc53 complex. Similar results were observed in complementary experiments, when Cdc4 was immunoprecipitated, and Cdc53 was probed for Fig. S5. Cdc4 is the F-box protein responsible for Gcn4 degradation. Therefore these observations run counter to the possibility that the change in Cdc4 levels, specifically a decrease upon shifting to MM + Met, contributes to Gcn4 stabilization under the same conditions. Collectively, these data diminish the possibility that methionine results in a decrease or absence of the SCF complex or F-box ubiquitin ligase that controls Gcn4 ubiquitination and degradation. These results suggest that methionine does not decrease the amounts of the ubiquitination complex known to ubiquitinate Gcn4.

### Phosphorylation at Thr^165^ is critical for Gcn4 degradation in the absence of methionine

The targeting of Gcn4 for ubiquitination by the SCF^Cdc4^ (and subsequent degradation) is regulated upstream by phosphorylation. In amino acid–replete conditions, the cyclin-dependent kinase Pho85, in coordination with the cyclin Pcl5, phosphorylates Gcn4 (44, 46) (Fig. 4*A*). Currently, Pho85 is the only kinase known to phosphorylate Gcn4 and regulate its stability. Downstream of this, phosphorylated Gcn4 becomes a substrate of the SCF^CDC4^ ubiquitination complex. Therefore, we asked whether methionine modulates the amounts of the Pho85–Pcl5 kinase complex. However, these two proteins were present at similar amounts in both methionine-replete and -depleted medium (Fig. S6), suggesting that kinase availability was not a limiting factor in regulating Gcn4 phosphorylation.

**Figure 5:**
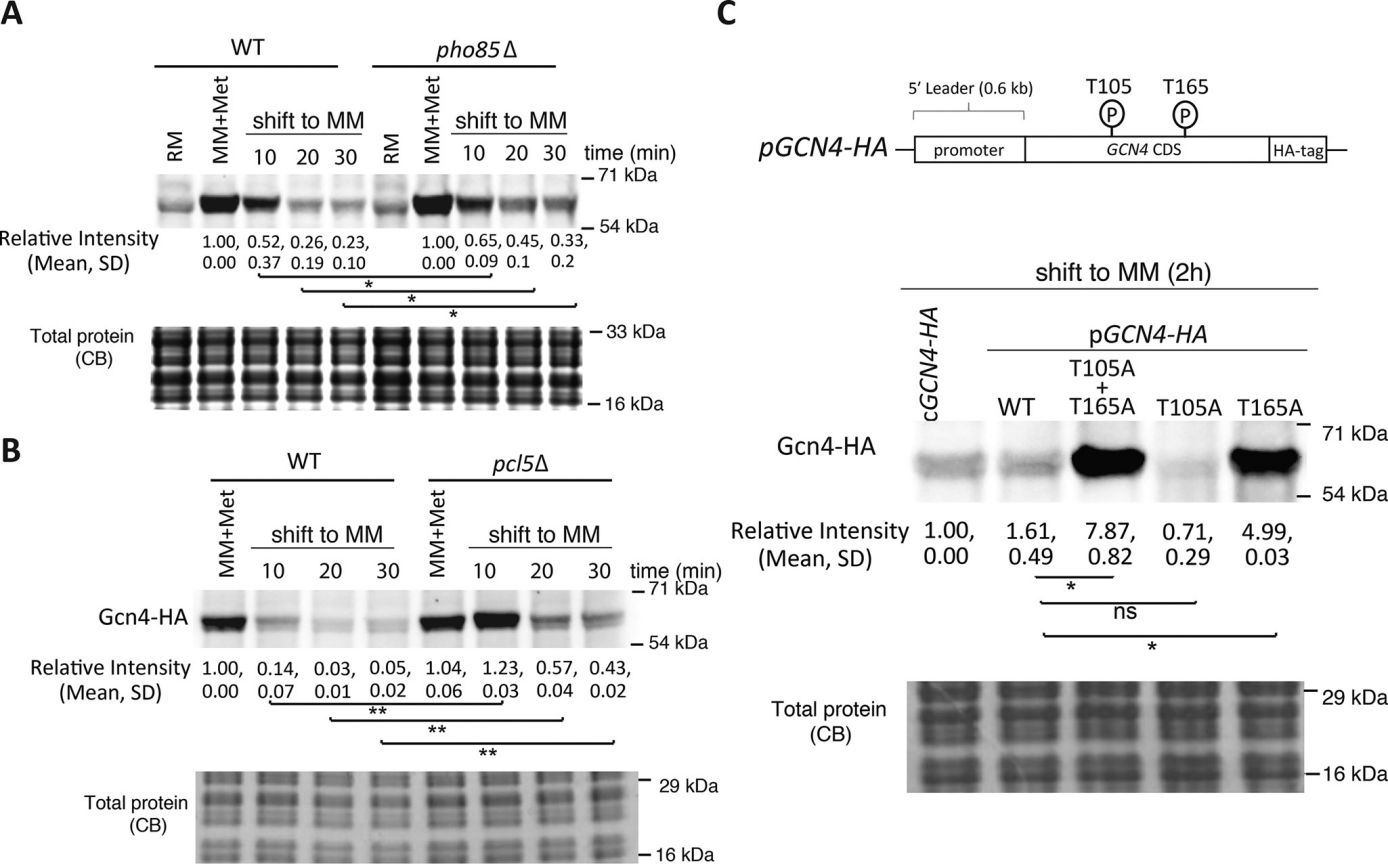
**Phosphorylation at Thr^165^ is critical for Gcn4 degradation in the absence of methionine.***A*, loss of Pho85-mediated phosphorylation stabilizes Gcn4 in MM. WT or *pho85*Δ cells in MM + Met were shifted to MM, and the amounts of Gcn4 were estimated by Western blotting. Relative Gcn4 amounts in the indicated conditions were quantified and are indicated *below* the blot (means ± S.D.). *, *p* < 0.05 (Student's *t* test). Each experiment was performed in biological replicates. Also see Fig. S6, S7, and S8. *B*, loss of Pcl5/Pho85-mediated phosphorylation maintains Gcn4 amounts when cells are shifted to MM. WT or *pcl5*Δ cells in MM + Met were shifted to MM, and the amounts of Gcn4 were estimated by Western blotting. Relative Gcn4 amounts in the indicated conditions were quantified and are indicated *below* the blot (means ± S.D.). *, *p* < 0.05; **, *p* < 0.01 (Student's *t* test). Each experiment was performed in biological replicates. *C*, Gcn4 phospho-mutants are stabilized in MM. The known phosphorylation residues (Thr^105^ and Thr^165^) related to the stability of Gcn4 were mutated to alanines, and the amounts of Gcn4 in WT cells or T105A/T165A mutant Gcn4 cells were compared. Relative Gcn4 amounts in the indicated conditions were quantified and are indicated *below* the blot (means ± S.D.). *, *p* < 0.05 (Student's *t* test). Each experiment was performed in biological replicates. *CB*, Coomassie Blue stain.

We next tested the importance of phosphorylation for Gcn4 stability in the absence or presence of methionine. Using *pho85*Δ and *pcl5*Δ cells, we first asked how the deletion of these proteins alters Gcn4 protein amounts. Note that the *pho85*Δ strain has a mild growth defect in RM, whereas the loss of Pcl5 has no obvious effect on growth (Fig. S7). The absence of either Pho85 or Pcl5 increased Gcn4 amounts in the absence of methionine (Fig. S8). We next directly investigated the kinetics of Gcn4 degradation upon removal of methionine. For this, the cells were shifted from RM to MM, and the Gcn4 amounts were measured over a very short time course in WT and *pho85*Δ or WT and *pcl5*Δ backgrounds (Fig. 5, *A* and *B*). This time course was chosen because data shown in Fig. 2*A*, as well prior literature (42), indicate that the *t*_1/2_ of Gcn4 is very short (∼5-15 min). Here, a rapid reduction in Gcn4 amounts was observed in WT cells (Fig. 5, *A* and *B*). These data are consistent with the rapid kinetics of Gcn4 degradation observed earlier in a different setup (Fig. 3*A*). In either *pho85*Δ or *pcl5*Δ cells, Gcn4 degradation was delayed, resulting in elevated protein levels after 30 min of shift in MM without methionine (Fig. 5, *A* and *B*). We do note that in the *pho85*Δ or *pcl5*Δ strains, whereas the kinetics of degradation is delayed when cells are shifted to MM, Gcn4 does appear to be degraded/decreased in amounts. This could suggest that (*a*) other kinases may compensate for the loss of Pho85 and/or (*b*) other proteins compensate for Pcl5 to sustain Pho85 kinase activity.

These data indicate that Pho85 kinase activity toward Gcn4 remains high in both methionine-replete and -depleted medium. However, this also suggests that the loss of this kinase could stabilize Gcn4 in the absence of supplemented methionine. Further, the canonical phosphorylation–ubiquitination pathway that controls the degradation of Gcn4 might be subject to regulation by methionine.

The Pho85/Pcl5 complex phosphorylates Gcn4 specifically at two independent sites, Thr^105^ and Thr^165^ (42, 44). To unambiguously study the phosphorylation-dependent regulation of Gcn4 stability by methionine, we next investigated the role of these two phosphorylation sites in mediating Gcn4 stability in MM. For this, a centromeric plasmid expressing native Gcn4 with a C-terminal HA tag, along with its endogenous promoter and regulatory regions, was used. In this plasmid, alanine mutations of the respective threonine residues (Thr^105^ and Thr^165^) individually or in combination were generated. These forms of Gcn4 were expressed in WT yeast. Subsequently, Gcn4 levels (in cells with these backgrounds) were estimated after shifting them from RM to MM. The WT Gcn4 was degraded in a manner indistinguishable from chromosomally tagged protein, confirming that this system fully recapitulates observations with native Gcn4 (Fig. 5*C*). Notably, the double-alanine mutant of Gcn4 was substantially stabilized in MM (where methionine was not supplemented). The Thr^105^ single mutant was degraded similar to WT, whereas the Thr^165^ mutant was stabilized (Fig. 5*C*). These data conclusively show that the phosphorylation of Gcn4 at Thr^165^ and subsequent ubiquitin-mediated degradation must be inhibited by methionine.

### The PP2A methyl transferase Ppm1 is required for methionine-mediated Gcn4 stabilization and function

These observations indicate that although Gcn4 ubiquitination and degradation remain high in the absence of methionine, Gcn4 is post-translationally stabilized by methionine. This degradation of Gcn4 depends on previously identified phosphorylation sites on this protein, but the known kinases themselves appear to be unaffected by methionine. Therefore, we wondered whether this methionine-mediated stabilization might instead be mediated by the selective activity of a protein phosphatase. If, in the presence of methionine, a phosphatase specifically targets Gcn4 for dephosphorylation, this would reduce phosphorylation marks on Gcn4 despite persistent kinase activity. Thus, such a mechanism could indirectly lead to Gcn4 stabilization in the abundant methionine.

**Figure 6:**
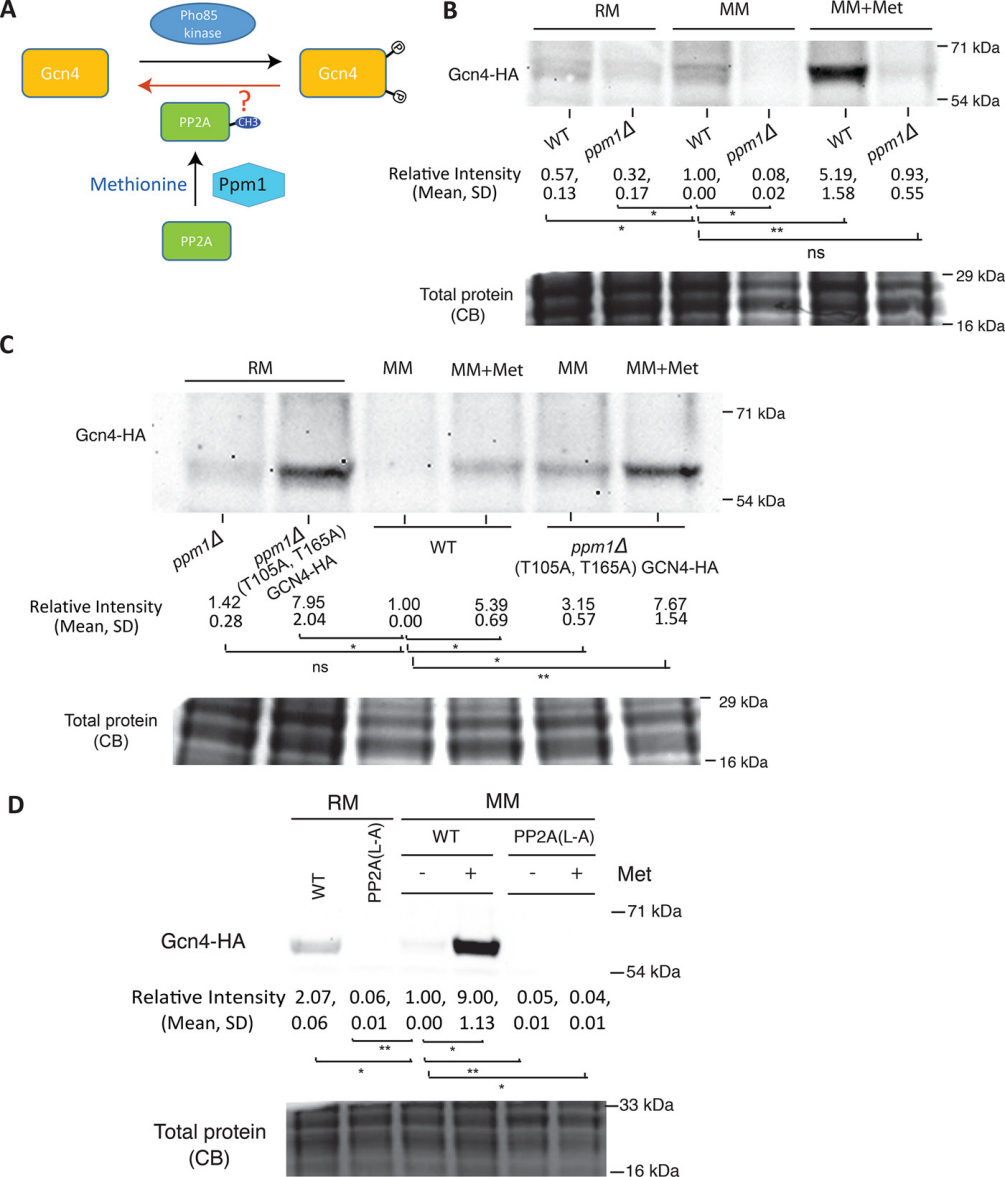
**The PP2A methyl transferase Ppm1 is required for methionine-dependent Gcn4 stabilization and function.***A*, a hypothetical mode by which Gcn4 phosphorylation might be regulated to determine Gcn4 stability. In methionine sufficiency, PP2A is carboxylmethylated by the methyltransferase Ppm1. If methylated PP2A targets Gcn4 for dephosphorylation, this will decrease Gcn4 ubiquitination and therefore degradation. *B*, loss of Ppm1 decreases Gcn4 amounts in MM + Met. The amounts of Gcn4 protein were compared in WT and *ppm1*Δ cells in both MM and MM + Met by Western blotting. Relative Gcn4 amounts in the indicated conditions were quantified and are indicated *below* the blot (means ± S.D.). *, *p* < 0.05; **, *p* < 0.01 (Student's *t* test). Each experiment was performed in biological replicates. *C*, Gcn4 phospho-mutants are stabilized in *ppm1*Δ cells in MM + Met. Gcn4 or Gcn4(T105A/T165A) protein amounts were compared in *ppm1*Δ cells in MM or MM + Met, as indicated. Relative Gcn4 amounts in the indicated conditions were quantified and are indicated *below* the blot (means ± S.D.). *, *p* < 0.05; **, *p* < 0.01 (Student's *t* test). Each experiment was performed in biological replicates. *D*, loss of PP2A methylation decreases Gcn4 amounts in MM + Met. The amounts of Gcn4 protein were compared in WT and PP2A(L→A) mutant cells in both MM and MM + Met by Western blotting. Relative Gcn4 amounts in the indicated conditions were quantified and are indicated *below* the blot (means ± S.D.). *, *p* < 0.05; **, *p* < 0.01 (Student's *t* test). Each experiment was performed in biological replicates. *CB*, Coomassie Blue stain; *ns*, not significant.

Interestingly, extensive evidence suggests important roles for a specific, methionine-responsive phosphatase in these conditions. Methionine strongly boosts the production of SAM, an important methyl donor (47), as established in these experimental conditions used (13, 48). Notably, methionine/SAM drives the methylation of the catalytic subunit of the protein phosphatase, PP2A (13, 17). The methyltransferase, Ppm1p (LCMT1 in mammals), specifically methylates PP2A at its C-terminal leucine residue (49, 50). Currently, Ppm1 is the only methyltransferase enzyme known to specifically methylate PP2A (50). Further, methylated PP2A (via Ppm1 activity) preferentially dephosphorylates specific substrates such as the TORC1 repressor, Npr2, in a methionine-responsive manner (48). This tunable substrate preference of PP2A made us ask whether methylated PP2A and Ppm1 activity might regulate Gcn4 stability (Fig. 6*A*). If this hypothesis is correct, an explicit prediction is that if PP2A methylation can be prevented, Gcn4 will be destabilized and degraded even in the presence of methionine.

To directly test this surmise, a strain lacking the Ppm1 methyltransferase was utilized. Gcn4 levels were measured after a shift to MM or MM + Met, in WT and *ppm1*Δ cells. After shifting cells to methionine-depleted medium (MM), Gcn4 amounts substantially decreased in both strains (Fig. 6*B*). This indicates that the Gcn4 degradation pathway remained functional in the mutant. Strikingly, in the *ppm1*Δ strain, substantially reduced amounts of Gcn4 was observed even in the presence of methionine, compared with WT cells (Fig. 6*B*). This indicates that Ppm1 activity is necessary to maintain high Gcn4 protein in the presence of methionine.

We next tested the importance of Gcn4 phosphorylation in this Ppm1-dependent degradation. We introduced the phosphorylation-insensitive point mutants of Gcn4 and asked whether this can rescue methionine-mediated Gcn4 stabilization in the absence of Ppm1. In *ppm1*Δ cells, the amounts of the alanine mutant form of Gcn4 remained high upon shift to MM + Met, whereas there was a reduction in amounts of WT protein (Fig. 6*C*). These data reveal that Ppm1 activity, which is required for PP2A carboxylmethylation, is critical for the stabilization of Gcn4. These results were further corroborated using a yeast strain in which the two isoforms of the PP2A catalytic subunit (Pph21 and Pph22) both have their respective C-terminal leucine residues mutated to alanines, rendering them insensitive to methylation (13). Consistent with the results observed with the *ppm1*Δ strain, in a PP2A methylation–deficient strain (PP2A-L→A), Gcn4 protein was not detectable in cells shifted to MM + Met (Fig. 6*D*). Taken together, these observations firmly establish that reversal of Gcn4 phosphorylation by methionine-sensitive Ppm1 and therefore PP2A phosphatase activity is both necessary and sufficient to rescue Gcn4 from ubiquitin-mediated degradation.

### Ppm1/PP2A methylation is critical for the Gcn4-mediated anabolic response in methionine-replete conditions

**Figure 7:**
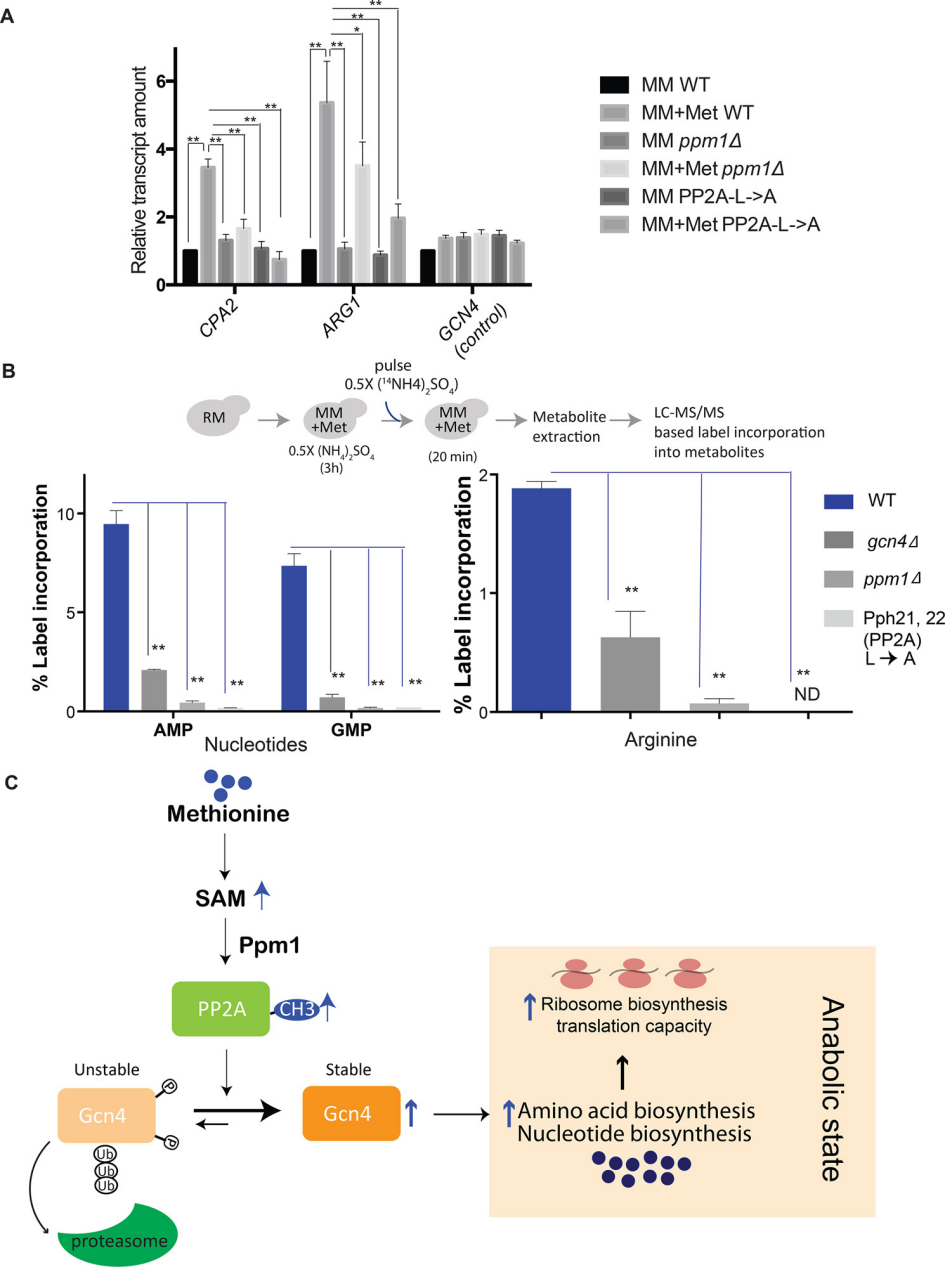
**Ppm1/PP2A methylation is necessary for the Gcn4 mediated anabolic response in methionine-replete conditions.***A*, relative transcript amounts (as measured using RT-qPCR) of Gcn4 target genes in cells from the indicated genetic background and grown in the indicated medium. Relative *GCN4* transcript amounts are also indicated (as a control). *ACT1* was used for normalization of the transcript abundance. The data show means ± S.D. from three biological replicates. *, *p* < 0.05; **, *p* < 0.01 (Student's *t* test). *B*, Gcn4-dependent anabolic precursor synthesis is absent in *ppm1*Δ and PP2A(L→A) cells. Quantitative, targeted LC–MS/MS based analysis of the *de novo* synthesis of Gcn4-dependent metabolites (AMP, GMP, and arginine). The indicated cells were shifted to MM + Met, pulsed with ^14^N-labeled ammonium sulfate, the metabolites were extracted, and label incorporation into the respective newly synthesized metabolites were estimated. The data shown are from two biological replicates (means ± S.D.). **, *p* < 0.01 (Student's *t* test). *ND*, not detectable/below detection limit. *C*, model illustrating how Gcn4 is stabilized by methionine via the action of Ppm1/PP2A methylation. In sufficient methionine, SAM amounts are high, and therefore the methyltransferase Ppm1 methylates the C-terminal leucine of PP2A. Methylated PP2A preferentially dephosphorylates Gcn4, thereby altering Gcn4 toward a less phosphorylated state. This decreased phosphorylation prevents the ubiquitination and therefore degradation of Gcn4, resulting in its accumulation. High Gcn4 amounts now drives amino acid and nucleotide biosynthesis, enabling cells to maintain an anabolic program.

Finally, we addressed the functional consequences of loss of PP2A methylation on Gcn4-mediated outputs, when methionine is abundant. For this, the relative transcript amounts of select direct Gcn4 targets (22) were compared in the following strains: WT, Pph21/22L→A, and *ppm1*Δ cells. As expected, in both the Pph21/22L→A and *ppm1*Δ cells, Gcn4 target transcripts were significantly reduced compared with WT cells (Fig. 7*A*).

We next directly analyzed the metabolic consequences of loss of PP2A methylation in the presence of methionine, using both Pph21/22L→A and *ppm1*Δ cells. This was addressed using direct, quantitative metabolic-flux based estimates of the *de novo* synthesis of key Gcn4 dependent metabolites by LC–MS. For this, ^15^N-labeled nitrogen (ammonium sulfate) was transiently pulsed, and the incorporation of this stable isotope of nitrogen into nucleotides or amino acids was quantitatively measured, using quantitative methods described earlier (14, 51) (also see the schematic illustration of Fig. 7*B*). Note that cells were collected after a brief pulse of adding the stable isotope (20 min for amino acids, 1 h for nucleotides), also controlling for growth and label incorporation, as standardized earlier (14, 51). This was done to ensure that the label incorporation faithfully reflected only the new synthesis of the respective metabolites and not steady-state levels of accumulated metabolites, as explained elsewhere (51). The results (Fig. 7*A*) unambiguously revealed that Pph21/22L→A, *ppm1*Δ and *gcn4*Δ cells all show comparable, poor label incorporation into these newly synthesized metabolites when compared with the WT cells. The Pph21/22L→A and *ppm1*Δ cells thus fully phenocopy the metabolic state of *gcn4*Δ cells in the presence of methionine. Taken together, these data show that the Ppm1-mediated methylation of PP2A controls the stability of Gcn4 and thereby the methionine-mediated anabolic program controlled by Gcn4.

## Discussion

In this study we elucidate a regulatory mechanism by which methionine induces Gcn4 to drive a growth program. In methionine-replete conditions, cells stabilize Gcn4, via the Ppm1-dependent methylation of the major protein phosphatase PP2A. The methylated form of PP2A preferentially shifts the pool of Gcn4 toward a dephosphorylated state. Dephosphorylated Gcn4 is protected from ubiquitination and subsequent proteasomal degradation and thereby increases in amounts. Ppm1 activity and PP2A carboxylmethylation-dependent stabilization of Gcn4 is essential for the sustained increase in amino acid and nucleotide synthesis in the presence of methionine. These findings therefore reveal a fundamental, metabolic function of PP2A, which is to increase the availability of key anabolic precursors (amino acids and nucleotides), upon sufficient availability of methionine (via its metabolite SAM). This is achieved by directly regulating the amounts of a metabolic master-regulatory transcription factor, Gcn4, as illustrated in a plausible model (Fig. 7*C*).

It becomes apparent that the mechanisms by which Gcn4 protein is regulated depends on context and the metabolic state of the cell. The best understood mechanism of Gcn4 (or ATF4) activation is via increased Gcn4 translation during amino acid starvation (24, 52). In conditions of acute amino acid starvation (including methionine starvation), cells activate the Gcn2 kinase and eIF2α phosphorylation, which reduces overall translation while increasing Gcn4 translation caused by the unique regulatory elements in Gcn4 mRNA. Through this mechanism, cells restore amino acid amounts during starvation. In direct contrast, here we illustrate an alternate mode of increasing Gcn4 activity when cells are in a growth state.

Here Gcn4 accumulates because of its increased stability in the presence of abundant methionine. Although the degradation of Gcn4 caused by phosphorylation-dependent ubiquitination is known (42, 44), the physiological contexts in which this mechanism is important are poorly studied. We show how, by using distinct signaling modules to increase Gcn4 stability, cells might activate this protein in a context-dependent manner. For example, if cells need to degrade Gcn4 in an amino acid–replete context, they can plausibly activate specific kinases that phosphorylate Gcn4, leading to its degradation. Conversely, alternate scenarios in which cells are in a growth state but still require Gcn4 function become easily possible. Methionine is a growth signal, activating both translation and the biosynthesis of metabolic precursors required for growth (11, 14, 22). In this context, Gcn4-dependent precursors critically sustain translation and this growth program (14, 22). By using a distinct, methionine-responsive phosphatase-based mechanism, the cells can now specifically increase Gcn4 amounts despite high rates of protein translation to sustain growth. By reducing the phosphorylation of Gcn4 in a methionine-dependent manner, Gcn4 can be selectively stabilized to drive anabolism.

A central concept emerging from this study is how metabolite responsive signaling can directly control metabolism. The molecular mechanisms by which methionine regulates signaling processes and thereby the downstream metabolic outputs are not yet clear. Although methionine (via SAM) activates the TORC1 complex (13, 15, 16), this mechanism does not explain how Gcn4 is activated nor how methionine controls the metabolic program. The discovery of the regulation of Gcn4 phosphorylation and stabilization via a methionine/SAM-specific modification of a phosphatase now provides a concise mechanism of action. The evidence for regulated PP2A activity and substrate selectivity via carboxylmethylation is now extensive (49, 50, 53, 54, 55, 56, 57, 58). The carboxylmethylation of PP2A, by Ppm1/LCMT1, alters the substrate-binding preference or specific activity of this phosphatase, as established in numerous systems (49, 53, 56, 57). What has been poorly explored are the contexts in which this methylation-dependent regulation plays a role. Here we establish that the methylated form of PP2A (which depends on the methyltransferase Ppm1) directly regulates cellular metabolic state in response to methionine/SAM, by increasing Gcn4 stability. Some alternate interpretations of these data remain possible. Thus far, the only known methyl transferase for PP2A is Ppm1 (50), but additional possibilities remain and potentially expand the scope of PP2A regulation. This includes the possibility that another methyltransferase might target PP2A for methylation and/or the presence of additional regulators of Gcn4 stability under these conditions.

These data also illustrate a context where the conditional, directed activity of a phosphatase and not the relevant kinase is critical in regulating cellular outcomes. In this context, the relevant kinase (Pho85 and its target cyclin Pcl5 and/or other as yet unidentified kinases) appear to be active in methionine-depleted and -replete conditions and continue to phosphorylate Gcn4. However, the conditional methylation of PP2A alters the balance of Gcn4 toward a dephosphorylated and therefore stabilized state. This study therefore completes an important conceptual loop: the metabolite (methionine) specifically regulates a signaling output that depends on the metabolite (the SAM-dependent methylation of PP2A). This leads to the increased dephosphorylation and stabilization of Gcn4, which eventually alters the metabolic state of the cell. This is an effective, directed means by which the cell can prioritize and manage available resources to commit to a growth state. We do note that the phosphorylation of Gcn4 by the kinases (which are methionine independent) might have more complex regulation, because the degradation of Gcn4 is not completely blocked upon the loss of Pcl5 (as seen in Fig. 5). This could involve alternate modes of activation of Pho85 or other kinases involved in this phosphorylation of threonines 105 and 165.

As introduced earlier, Gcn4 is an evolutionarily conserved regulator of amino acid biosynthesis that has canonically been studied in the context of starvation. In contrast, during a methionine-driven growth program, Gcn4 activity fuels the synthesis of required biosynthetic precursors to drive growth. Interestingly, such scenarios are also observed in several tumors, in which increasing evidence shows that the mammalian ortholog of Gcn4 (ATF4) is induced and assists in tumor proliferation (59, 60, 61, 62). This is likely by providing necessary metabolic intermediates that enable these cells to grow. The mechanisms of induction of ATF4 in these contexts remain very poorly understood. It is conceivable to suggest that the mechanism observed in this study is plausible in such disease-relevant contexts of high growth.

We can now summarize a unifying model for how methionine launches a multipronged transcriptional and metabolic program to drive growth, based on distinct studies (11). The key elements in this transformation include: tRNA thiolation–mediated routing of carbon-flux toward the PPP, as well as maintaining overall metabolic homeostasis (41), Gcn4-mediated increased biosynthesis of amino acids and nucleotides (14, 22), maintaining sufficient translation capacity (22), balancing phospholipid and histone methylation (17), and the SAM-mediated activation of the TORC1 pathway (13, 15, 16). In this methionine-induced growth program, the roles of the methyltransferase Ppm1 and methylation-dependent PP2A activity appear to be central. Methylated PP2A activates the TORC1 pathway (13) and global histone methylation (17, 63) and also controls anabolic precursor synthesis via the activation of Gcn4 (this study).

Many of these processes appear to be evolutionarily conserved and observed in yeast and mammals. This includes the degradation of ATF4 via a phosphorylation-dependent interaction with a ubiquitin ligase (64). It will therefore be interesting to evaluate the roles of signaling outputs that depend on methylated PP2A, in relevant contexts of growth regulation in healthy and diseased tissue. The SAM-sensitive methylation of PP2A could be a universal mode by which cells directly sense this key metabolite (methionine), dictate global phosphorylation status, and correspondingly regulate metabolism to drive growth programs.

## Materials and methods

### Yeast strains and growth media

A fully prototrophic CEN.PK strain (65) (referred to as WT) was used in all experiments. Strains with gene deletions or chromosomally tagged proteins (at the C terminus) were made using methods and reagents described elsewhere (66). The strains used in this study are listed in Table 1.

**Table 1:** Strains used in this study

Strain background	Genotype	Reference
CEN.PK	MAT a (full prototroph)	Ref. 65
CEN.PK	MAT a *GCN4-HA-KanMX*	Ref. 14
CEN.PK	MAT a *gcn4*Δ::*NAT*	Ref. 14
CEN.PK	MAT a *SUI2-FLAG-NAT* (eIF2α)	This study
CEN.PK	MAT a *SUI2-FLAG-NAT GCN4-HA-KanMX*	This study
CEN.PK	MAT a *SUI2-FLAG-NAT GCN4-HA-KanMX gcn2*Δ::*HYG*	This study
CEN.PK	MAT a *GCN4-HA-KanMX pdr5*Δ::*HYG*	This study
CEN.PK	MAT a *CDC4-HA*::*KanMX*	This study
CEN.PK	MAT a *CDC4-HA*::*KanMX CDC53-FLAG*::*Hyg*	This study
CEN.PK	MAT a *pho85*Δ::*NAT gcn4*Δ::*KanMX*	This study
CEN.PK	MAT a *pcl5*Δ::*NAT gcn4*Δ::*KanMX*	This study
CEN.PK	MAT a *ppm1*Δ::*NAT gcn4*Δ::*Hyg*	This study
CEN.PK	MAT a *ppm1*Δ::*NAT GCN4-HA*::*KanMX*	This study
CEN.PK	MAT a *pdr5*Δ::*Hyg ppm1*Δ::*NAT GCN4-HA*::*KanMX*	This study
CEN.PK	Pph21/22L→A	Ref. 13

The growth media used in this study are RM (1% yeast extract, 2% peptone and 2% lactate) and MM (0.17% yeast nitrogen base without amino acids, 0.5% ammonium sulfate and 2% lactate). All amino acids were supplemented at 2 mm. Non-SAAs refers to the mixture of all standard amino acids (2 mm each) except methionine, cysteine, and tyrosine, as described previously (14). The indicated strains were grown in RM with repeated dilutions (∼36 h), and the culture in the log phase (absorbance at 600 nm of ∼1.2) was subsequently switched to MM, with or without the addition of the indicated amino acids.

### Polysome analysis

The cells (50 *A*_600_) were treated with cycloheximide (100 μg/ml) for 15 min before harvesting in ice-filled, cold centrifuge bottles. The pellet obtained after centrifugation (8000 × *g*, 5 min, 4 °C) was washed once and then resuspended in 0.5 ml of lysis buffer (10 mm Tris-HCl, pH 7, 0.1 m NaCl, 30 mm MgCl_2_, 1 unit/μl Ribolock RNase inhibitor, and 80 μg/ml cycloheximide) with chilled, baked acid-washed glass beads. After bead beating, 0.5 ml of lysis buffer was added, the crude extract obtained was centrifuged (18,407 × *g*, 10 min, 4 °C), and the supernatant (200 μl) containing polysomes was fractionated on a sucrose gradient (7–47% sucrose; containing 20 mm Tris-HCl, pH 7, 140 mm KCl, 5 mm MgCl_2_, 1 mm DTT, and 50 μg/ml cycloheximide) by ultracentrifugation (35,000 rpm, 3 h, 4 °C). The polysome profile was obtained on an ISCO fractionator, wherein the absorbance at 254 nm was traced. For polysome:monosome ratio calculation, the area under the curve was quantified using the ImageJ software (Rasband, ImageJ, National Institutes of Health, Bethesda, Maryland, USA, RRID:SCR_003070, 1997–2016).

### Western blots

Approximately ten *A*_600_ cells (∼10 ml of cells at *A*_600_ ∼1) were collected from the respective cultures, pelleted, and flash-frozen in liquid nitrogen until further use. The cells were resuspended in 400 μl of 10% TCA and lysed by bead beating three times: 30 s of beating and then 1 min of cooling on ice. The precipitates were collected by centrifugation, resuspended in 400 μl of SDS-glycerol buffer (7.3% SDS, 29.1% glycerol and 83.3 mm Tris base), and heated at 100 °C for 10 min. The supernatant after centrifugation was treated as the crude total protein extract. Protein concentrations from extracts were estimated using a bicinchoninic acid assay (Thermo Scientific). Equal protein amounts of each sample was resolved on 4–12% Bis-Tris gels (Invitrogen). Coomassie Blue–stained gels were used as loading controls. Western blots were developed using antibodies against the respective tags. The following primary antibodies were used: monoclonal FLAG M2 (F3165, Sigma), HA (12CA5, Roche), and phosphor-eIF2α Ser51 (9721, Cell Signaling). Horseradish peroxidase–conjugated secondary antibodies (mouse and rabbit) were obtained from Sigma. The molecular weight markers used were 2661 (Thermo Scientific) and PG-P MT2922 (Genetix). For Western blotting, standard enhanced chemiluminescence reagents (GE Healthcare) were used. ImageJ was used for quantification.

### GCN4-Luc constructs

A series of constructs having variations of the *GCN4* promoter were generated as shown in Fig. S1 and Fig. 2*B*, based on a validated *GCN4* reporter from an earlier study (41). The luciferase reporter gene was synthesized into a pGL3basic vector (Promega) and subcloned in EcoRI and XhoI sites of the CEN/ARS, single-copy plasmid p417-TEF-KAN plasmid (modified from p417-CYC, MoBiTech, Germany). The resulting plasmid was utilized for inserting different synthesized fragments (GeneArt), having indicated point mutations in the promoter elements and the first 55 codons of *GCN4* ORF, using SacI and EcoRI sites just upstream of the luciferase gene. Thus, in all the constructs, *GCN4* promoter was followed by the *GCN4* ORF (encoding the first 55 amino acids) fused to the firefly luciferase coding sequence. The luciferase activity was measured using a luciferase assay kit (Promega, E1500).

### Gcn4 phospho-mutants

The complete uORF reading frame of *GCN4* and along with the Gcn4 coding sequence, followed by a 6× HA epitope tag at the C terminus, followed by an Adh1 terminator sequence, were amplified from genomic DNA obtained from the Gcn4-HA strain. This was cloned into a CEN.ARS plasmid (p417-cyc), where the plasmid promoter sequence was also replaced with the complete *GCN4* upstream regulatory region. This resulted in a plasmid that can express full-length Gcn4 with a C-terminal 6× HA epitope tag, with fully endogenous promoter and ORF regulatory regions included. This construct sequence was confirmed by sequencing. The identified phosphorylation site residues (Thr^105^ and Thr^165^) were mutated to alanine residues individually or in combination, by standard site-directed mutagenesis.

### RNA isolation and RT-qPCR

Total RNA from yeast cells was extracted using a hot acid phenol method (67). SuperScript II reverse transcriptase (Invitrogen) was used for the reverse transcription of the total RNA (1 μg). Quantitative PCR was performed with the synthesized cDNA using SYBR Green (Thermo Scientific) and specific primers. *ACT1* was used for normalization of the transcript abundance. The primers used were *GCN4*, TGCTTACAACCGCAAACAGC and GCACGTTTTAGAGCAGCAGG; *ACT1*, TCGTTCCAATTTACGCTGGTT and CGGCCAAATCGATTCTCAA; *ARG1*, AAGGCCAAGCCATGGTCTAC and TCCACATGTCCTTTGGTGGG; and *CPA2*, GGTTAGGCTCAGGTTTCGCT and GAATTTGTGGGGCCAACGAC.

For the RT-qPCR on polysome fractions, the first RNA was precipitated from individual fractions, quantitated, and then used for the RT-qPCR. Fractional abundance was calculated based on the assumption that all fractions sum up to have abundance of 1.

### Metabolite extractions and measurements by LC–MS/MS

For detecting ^15^N-label incorporation in amino acids and nucleotides, [^15^N]ammonium sulfate with all nitrogens labeled (NLM-713-PK, Cambridge Isotope Laboratories) was used. At the end of the incubation with the spiked label, the cells were rapidly harvested, and metabolite was extracted as described earlier (14, 51). Briefly, the metabolites were measured using the LC–MS methods described earlier (51). Standards were used for developing multiple reaction monitoring methods on a Sciex QTRAP 6500 (used in triple-quadrupole mode). All measurements were done in positive polarity mode. For this, metabolites were separated using a Synergi 4 μ Fusion-RP 80A column (150 × 4.6 mm, Phenomenex) on Agilent's 1290 infinity series UHPLC system coupled to mass spectrometer. The buffers used for separation were buffer A: 99.9% H_2_O, 0.1% formic acid and buffer B: 99.9% methanol, 0.1% formic acid (flow rate, 0.4 ml/min; *T* = 0 min, 0% B; *T* = 3 min, 5% B; *T* = 10 min, 60% B; *T* = 10.1 min, 80% B; *T* = 12 min, 80% B; *T* = 14 min, 5% B; *T* = 15 min, 0% B; *T* = 20 min, stop). The area under each peak was calculated using the AB SCIEX MultiQuant software 3.0.1

For all the nucleotide measurements, release of the nitrogen base was monitored. All the mass transitions of parent/product masses measured for the different metabolites are listed in Table 2.

**Table 2:** Mass transitions used for the ^15^N label incorporation LC/MS/MS experiment

Molecule of interest	Formula	Parent/product (positive polarity)	Comment
^15^N_AMP_1		349/137	
^15^N_AMP_2		350/138	
^15^N_AMP_3		351/139	
^15^N_AMP_4		352/140	
^15^N_AMP_5		353/141	
GMP	C_10_H_14_N_5_O_8_P	364/152	
^15^N_GMP_1		365/153	
^15^N_GMP_2		366/154	
^15^N_GMP_3		367/155	
^15^N_GMP_4		368/156	
^15^N_GMP_5		369/157	
Arg	C_6_H_14_N_4_O_2_	175.2/60	Product has only one N
^15^N_Arg_1		176.2/61	
^15^N_Arg_2		177.2/61	
^15^N_Arg_3		178.2/61	
^15^N_Arg_4		179.2/61	
AMP	C_10_H_14_N_5_O_7_P	348/136	Product has all N

## Data availability

All data are available within the article.
