# Supplementary material for: Methylated PP2A stabilizes Gcn4 to enable a methionine-induced anabolic program
Source: J Biol Chem. 2021 Jan 13;295(52):18390–405. doi: 10.1074/jbc.RA120.014248 (PMC7939465; doi:10.1074/jbc.RA120.014248)
Supplement: Supplementary file 1 [file mmc1.pdf]

Supporting Information

Methylated PP2A stabilizes Gcn4 (ATF4) to enable a methionine-induced anabolic program

Adhish S. Walvekar[1,3#], Ganesh Kadamur[1#], Sreesa Sreedharan[1,2], Ritu Gupta[1], Rajalakshmi Srinivasan[1] and Sunil Laxman[1*]

[1]Institute for Stem Cell Science and Regenerative Medicine (inStem)

GKVK Post Bellary Road

Bangalore 560065

[2]School of Chemical and Biotechnology

SASTRA University

Tanjavur 613401

[3] Present address: Luxembourg Centre for Systems Biomedicine, University of Luxembourg, L-4367 Belvaux, Luxembourg

[#] these authors contributed equally to this manuscript

*Corresponding author: Sunil Laxman

Email: sunil@instem.res.in

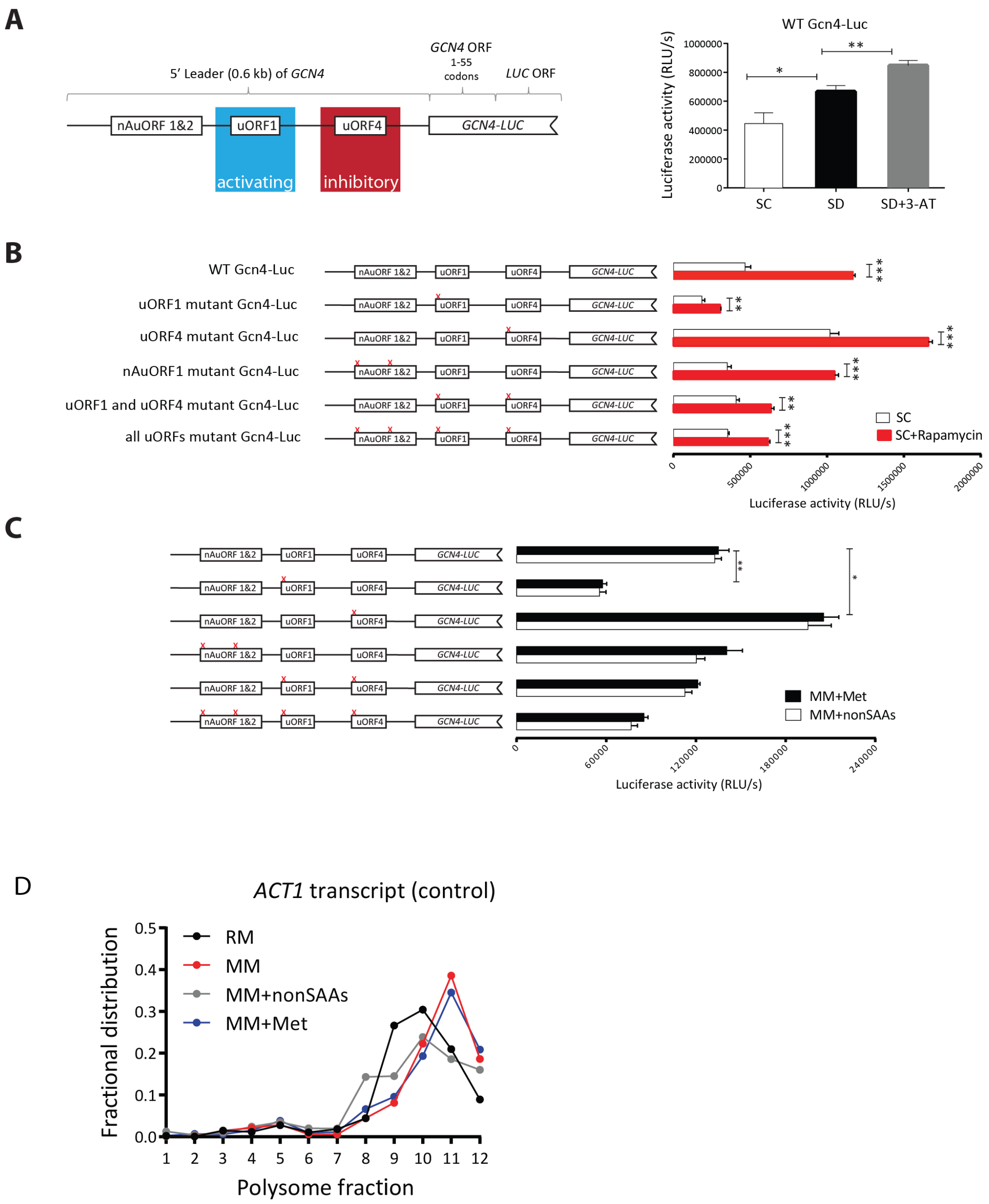

Figure S1

Figure S1: Gcn4 translation in MM, MM+nonSAAs and MM+Met, based on reporter activity or polysome fractions.

A) Schematic illustrating the design of a luciferase based reporter to estimate the translation of *GCN4* transcripts, and validation of the reporter for *GCN4* translation in different medium.
B) Relative *GCN4* translation in complete minimal glucose medium with all amino acids (SC), or the same medium with rapamycin added to induce *GCN4* translation, as measured using a series of luciferase-based *GCN4* translation reporters. The relative luciferase activity is shown on the y-axis of the plots,

while the different reporters used are illustrated on the left. The data shown are from three biological replicates, mean ± SD. * $p<0.05$ (Student's t-test).
C) Relative *GCN4* translation in MM+nonSAAs or MM+Met, as measured using a series of luciferase-based *GCN4* translation reporters. The relative luciferase activity is shown on the y-axis of the plots, while the different reporters used are illustrated on the left. The data shown are from three biological replicates, mean ± SD. * $p<0.05$ (Student's t-test). MM+nonSAAs is MM supplemented with all non-sulfur containing amino acids (i.e. excluding methionine and cysteine).
D) *ACT1* transcript amounts in different polysome fractions, obtained from cells grown in RM, MM or MM+met. Transcripts were measured using standard, quantitative RT-qPCR approaches.

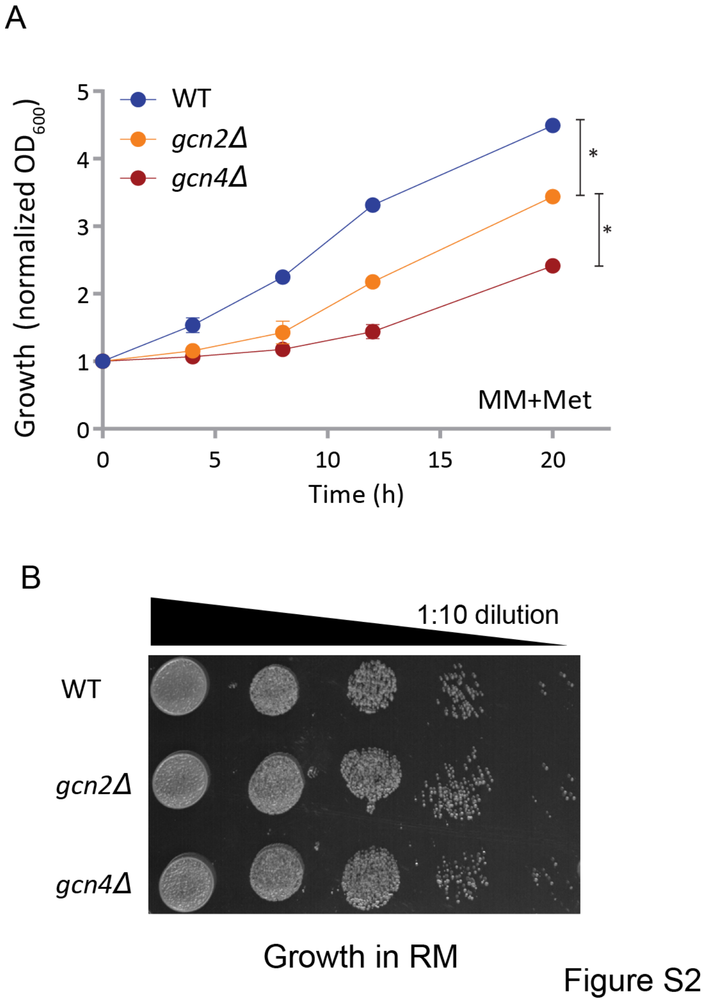

Figure S2: Comparison of growth in MM+Met

A) Growth curves of wild type (WT), *gcn2*Δ and *gcn4*Δ cells, shifted from RM to MM+Met. Data are from biological triplicates, showing mean±SD, * indicates $p<0.05$ (Student's t-test).

B) Serial-dilution based assay comparing the growth of WT, *gcn2*Δ and *gcn4*Δ cells in RM (control).

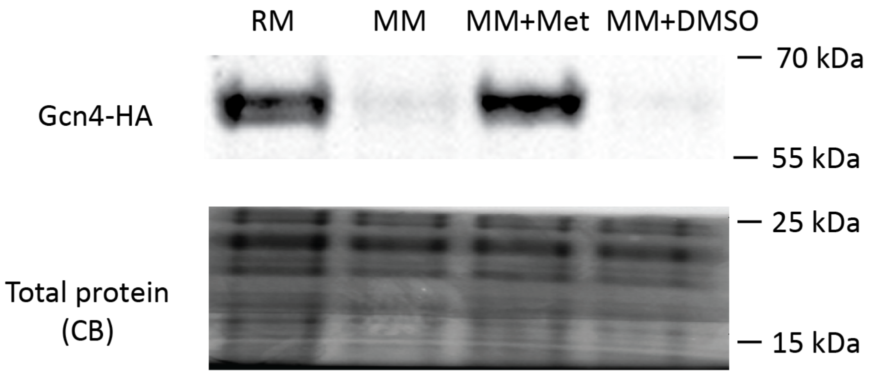

Figure S3

Figure S3: Gcn4 expression in MM with the addition of DMSO

Vector control (DMSO addition) does not induce Gcn4 in MM. DMSO alone was added to WT cells in MM, at the same amount as that for MG-132 with DMSO.

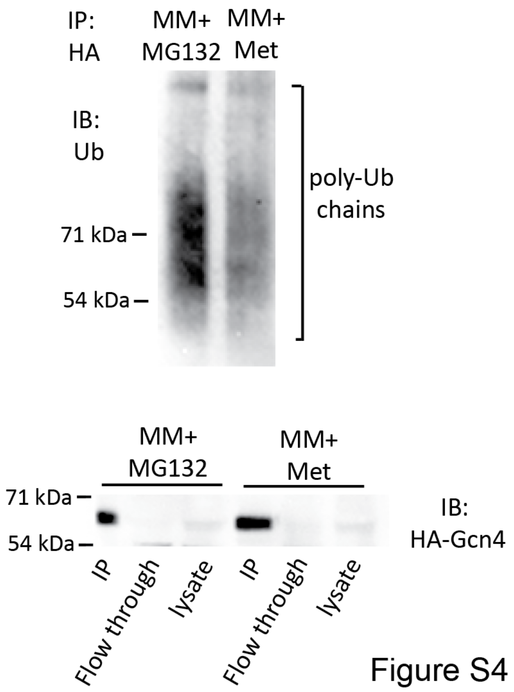

Figure S4: Gcn4 ubiquitination in MM or MM+Met
Biological replicate of Gcn4 ubiquitination in the presence or absence of methionine (also see Figure 3E). Cells growing in MM (with MG132 added) or MM+Met were collected, and Gcn4 was immunopurified. The immunopurified Gcn4 was resolved on SDS-PAGE gels, and poly-ubiquitin chains were detected by Western blotting using an ubiquitin-specific antibody. Cells in MM+Met show substantially decreased polyubiquitin bands. IB: immunoblot, IP: immunopurification, CB: Coomassie blue stain.

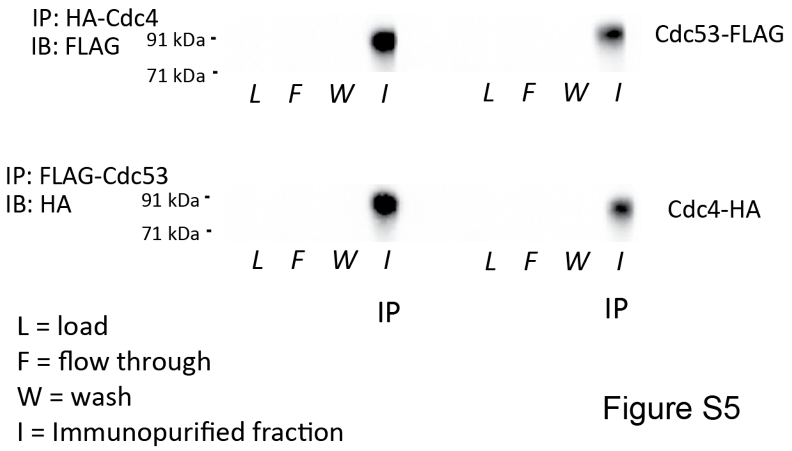

Figure S5: Interaction of Cdc53 and Cdc4, and the formation of the SCF^{CDC4} complex.

Cells in MM or MM+Met were collected, Cdc4 (HA) was immunoprecipitated and the samples subject to HA immunoblots to assess the association between Cdc4 and Skp1-Cdc53 complex. Also see Figure 4C.

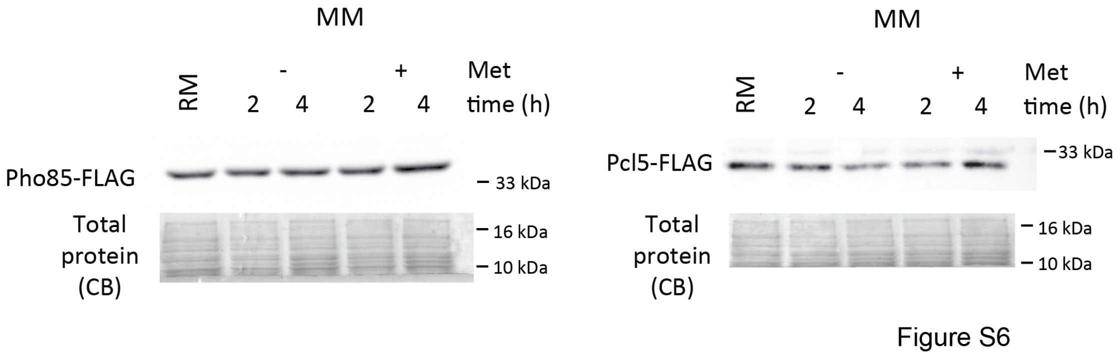

Figure S6: Qualitative assessment of the Pho85 and Pcl5 proteins, which comprise the Pho85-Pcl5 kinase complexes, in MM and MM+Met.
A) Amounts of Pho85 in MM or MM+Met. Cells in RM were shifted to MM or MM+Met and Pho85 amounts were measured by Western blotting (anti-FLAG).
B) Amounts of Pcl5 in MM or MM+Met. Cells in RM were shifted to MM or MM+Met and Pcl5 were detected by Western blotting (anti-FLAG).

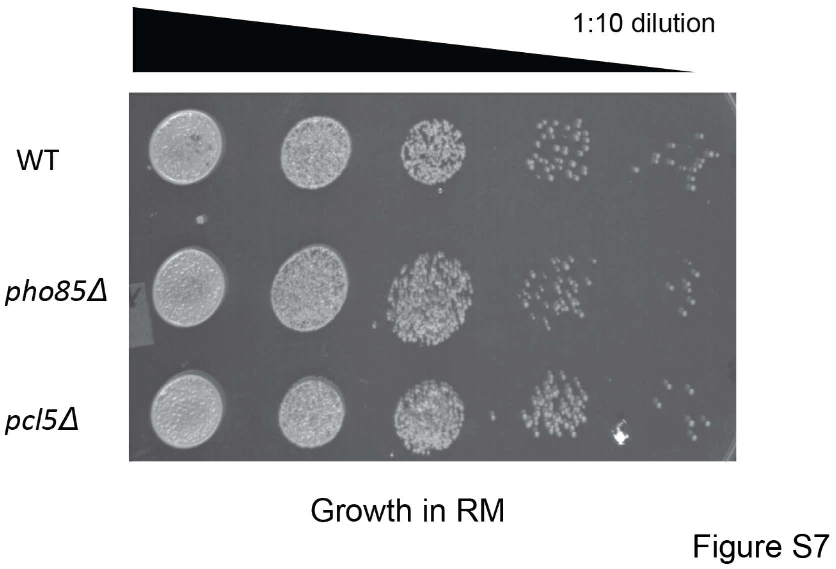

Figure S7: Relative growth of WT, *pho85*Δ and *pcl5*Δ cells
Serial dilution based growth assays of WT, *pho85*Δ and *pcl5*Δ cells (all in a Gcn4-HA background), on solid agar RM.

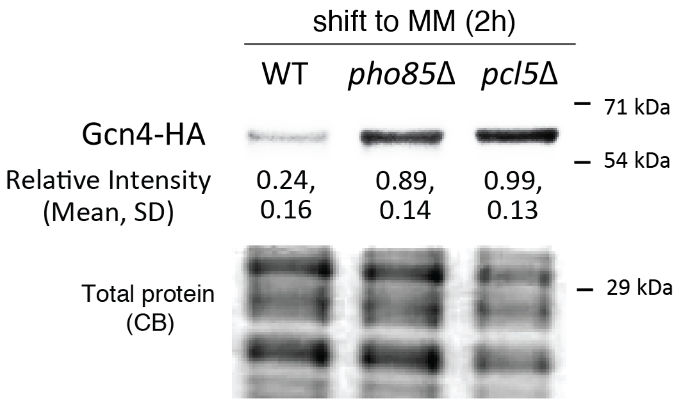

Figure S8

Figure S8: Loss of phosphorylation of Gcn4 mediated by Pho85 or Pcl5 results in increased Gcn4 stability.

The blot shows Gcn4 protein observed in WT, *pho85Δ*, or *pcl5Δ* cells when shifted to MM from MM+Met. Also see Figure 4A and 4B.
